# Bark and leaf chlorophyll fluorescence are linked to wood structural changes in *Eucalyptus saligna*

**DOI:** 10.1093/aobpla/plt057

**Published:** 2014-02-03

**Authors:** Denise Johnstone, Michael Tausz, Gregory Moore, Marc Nicolas

**Affiliations:** 1 Department of Resource Management and Geography, University of Melbourne, Burnley Campus, Richmond 3012, Australia; 2 Department of Forest and Ecosystem Science, University of Melbourne, Creswick Campus, Creswick 3363, Australia; 3 Department of Agriculture and Food Systems, University of Melbourne, Parkville Campus, Parkville 3010, Australia

**Keywords:** Bark, chlorophyll fluorescence, photosynthesis, stress physiology, wood decay, wood structure.

## Abstract

Wood structure and wood anatomy are usually considered to be largely independent of the physiological processes that govern tree growth. This paper reports a statistical relationship between leaf and bark chlorophyll fluorescence and wood density. A relationship between leaf and bark chlorophyll fluorescence and the quantity of wood decay in a tree is also described. There was a statistically significant relationship between the leaf chlorophyll fluorescence parameter *F*_v_/*F*_m_ and wood density and the quantity of wood decay in summer, but not in spring or autumn. Leaf chlorophyll fluorescence at 0.05 ms (the O step) could predict the quantity of wood decay in trees in spring. Bark chlorophyll fluorescence could predict wood density in spring using the *F*_v_/*F*_m_ parameter, but not in summer or autumn. There was a consistent statistical relationship in spring, summer and autumn between the bark chlorophyll fluorescence parameter *F*_v_/*F*_m_ and wood decay. This study indicates a relationship between chlorophyll fluorescence and wood structural changes, particularly with bark chlorenchyma.

## Abstract (precis)

The efficiency of leaf photosynthesis has been measured using a technique called chlorophyll fluorescence. It is not widely known that in addition to leaf photosynthesis the bark of certain “smooth barked” trees can photosynthesize. In this paper we use chlorophyll fluorescence to measure the efficiency of bark photosynthesis. In this way we are able to compare the amount of wood decay in a tree with bark photosynthetic efficiency using chlorophyll fluorescence. The link between bark photosynthesis and wood decay discovered in this work has not been explored before.

## Introduction

Tree physiology and wood structure and anatomy are often considered to be independent, as wood occurs primarily in what is sometimes described as the non-functioning heartwood of the tree (Zweifel *et al.* 2006). On the other hand, wood as a tissue (i.e. the secondary xylem of trees) determines long-distance water transport in trees. During water transport, if xylem vessels are under water stress, air bubbles in the xylem can expand due to tension, a process known as cavitation (Hacke *et al.* 2001; Taiz and Zeiger 2010). Once a xylem vessel cavitates it fills with water vapour and then forms an embolism in quick succession, slowing xylem hydraulic conductivity (Tyree and Sperry 1989). Therefore, wood density is increasingly being measured in conjunction with water-use properties, as low stem wood density can make angiosperms more vulnerable to cavitation, especially during drought (Hacke *et al.* 2001; Holste *et al.* 2006; Bobich *et al.* 2010). However, conifers do not necessarily follow this pattern as their xylem conduits are shorter and narrower. In a study of *Picea abies* (Norway spruce), wood density was unrelated to xylem cavitation (Rosner *et al.* 2007). The relationship between wood decay and physiological measurements not directly related to water use has rarely been assessed. Wood structural changes are frequently caused by wood decay organisms (Rayner and Boddy 1988). Decayed wood shows decreased density as a result of degradation by fungi or bacteria (Harris *et al.* 2004). Weight loss or dry weight is a common means by which to evaluate wood decay, particularly in the early stages of decay (Wilcox 1978; Pandey and Pitman 2003; Wei *et al.* 2010). Despite ongoing methodological difficulties, wood decay can be quantified by a variety of methods, such as with devices using electrical conductivity, drilling resistance, core sampling or acoustic methods (Johnstone *et al.* 2010*a*). It appears logical that wood decay, leading to decreased wood density, can affect tree water transport and, consequently, canopy physiology, mainly under periods of increased demand on water transport. Because wood decay involves invading organisms such as fungi or bacteria, it may also be speculated that biochemical changes (e.g. defence reactions) can affect the physiological function of other tissues.

Trees have chlorenchyma, i.e. photosynthetically active tissue, in their bark below the rhytidomal or outer peridermal layers (Strain and Johnson 1963; Pfanz *et al.* 2002). Such cortical or peridermal chlorenchyma is able to utilize CO_2_ from gaseous xylem efflux and from mitochondrial respiration to photosynthesize (Wittmann *et al.* 2006; Pfanz 2008). Bark photosynthesis can be strongly shade adapted, particularly in deciduous trees (Pfanz *et al.* 2002; Damesin 2003; Manetas 2004). *Eucalyptus globulus* bark behaved as a shade leaf in a study by Eyles *et al.* (2009); however, Tausz *et al.* (2005) found that parts of sun-exposed *Eucalyptus nitens* bark had photosynthetic pigments of similar quantity and composition to that of sun leaves. Bark photosynthetic activity in stems is generally lower than in the leaves of broadleaf trees such as *Betula pendula*, *Quercus robur* and *Fagus sylvatica*, but it could be a way of improving the carbon balance of stems, particularly where water is limiting (Wittmann and Pfanz 2008*b*).

Chlorophyll fluorescence (CF) is an excellent tool to assess the physiological state of photosynthetic tissues (Govindjee 2004). *F*_v_/*F*_m_ is the most commonly cited CF parameter, where *F*_v_ is the difference between maximum (*F*_m_) and minimum (*F*_o_) fluorescence (Maxwell and Johnson 2000). *F*_v_/*F*_m_ is the theoretical measure of the quantum efficiency of photosystem II (PSII) if all the PSII reaction centres are open (Maxwell and Johnson 2000). The average *F*_v_/*F*_m_ value for healthy tissues is believed to be around 0.83 (Bjorkman and Demmig 1987; Johnson *et al.* 1993). Decreased values indicating reduced maximum quantum efficiency commonly occur upon impact of environmental stress. *F*_v_/*F*_m_ is therefore commonly used to assess stress impacts on plants (Maxwell and Johnson 2000).

The analysis of the intermediate data points of the fast fluorescence rise (i.e. the determination of *F*_m_ in the calculation of *F*_v_/*F*_m_) is called the O–J–I–P polyphasic fast fluorescence rise analysis or the O–K–J–I–P polyphasic fast fluorescence rise analysis (Susplugas *et al.* 2000; Strasser and Stirbet 2001; Govindjee 2004; Strasser *et al.* 2004; Percival 2005). The phases are O at the origin (0.05 ms), K at ∼0.2 ms, J at ∼2 ms, I at ∼20 ms and P at ∼200 ms, depending on the curve (Strasser and Stirbet 2001). O or *F*_o_ fluorescence is measured when all the plastoquinone Q_A_ electron carrier molecules are in their oxidized state (Krause and Weis 1984; Percival 2005). The K step, not apparent in all cases, may be the result of an imbalance in electron flow coming to the reaction centre from PSII in some species of plants (Strasser *et al.* 2004). The O–J phase is believed to represent the reduction of the Q_A_ molecule from Q_A_ to Q_A_^−^ (Hsu and Leu 2003; Strasser *et al.* 2004; Percival 2005). J–I may be fluorescence from the abaxial layer of the sample in some plants (Hsu and Leu 2003), or both the J–I and I–P phases could reflect the existence of fast and slow reducing plastoquinone centres (Percival 2005). P or *F*_m_ occurs when all the plastoquinone Q_A_ electron carrier molecules are in their reduced state (Krause and Weis 1984; Percival 2005). The characteristics of the fast fluorescence rise also change upon stress impact, and are therefore used to assess stress impacts on plants.

There is evidence that leaf photosynthetic capacity and the hydraulic properties of tree stems are related (Brodribb and Feild 2000; Brodribb *et al.* 2007), yet any direct relationship between wood properties or wood decay and photosynthetic properties has rarely been examined. The symptoms of ‘esca’ disease in *Vitis vinifera* (grapevines) and CF parameters have been linked (Christen *et al.* 2007). Esca disease infects the xylem and causes the white rot decay and/or necrosis of woody tissues and, subsequently, wilting of the leaves. However, no investigations using tree species prior to the current study have attempted to relate photosynthetic properties to wood decay.

In a previous study, the authors investigated a relationship between crown condition and leaf and bark CF (Johnstone *et al.* 2012). There was little evidence to support a relationship between leaf CF and crown condition. On the other hand, there was a strong relationship between bark CF and crown condition. The current study uses the leaf and bark CF data from the above-mentioned study, but compares it with wood density and wood decay. In this study, the relationship between CF and wood structural properties is examined, rather than CF and growth parameters.

The current study investigated plantation-grown *Eucalyptus saligna* trees exhibiting a range of wood decay from virtually none to moderately decayed. We chose trees already decayed as inducing decay in trees can be a slow process, dependent on tree species and the causal agent of decay (Schwarze 2008). Trees were chosen to represent the best possible range of decay under otherwise uniform conditions. We examined the relationships between wood decay and density and CF in leaf and bark tissues to test the following hypotheses: (i) increasing wood decay is related to stress symptoms in leaves, particularly in summer when demand on xylem water transport is greatest, and (ii) increasing wood decay is related to stress symptoms in bark chlorenchyma.

## Methods

The trees used in this study were *E. saligna* (Bateman's Bay). They were ∼20 years old in 2008, between 17 and 27 m high, and with diameters at 1.3 m of between 142 and 318 mm. The 36 selected trees were part of a larger species/provenance study covering a total area of ∼10 ha in a eucalypt plantation at Tostaree in rural Victoria, Australia (latitude 37°47′; longitude 148°11′). Sample trees were chosen to represent a range of wood decay and excluded any break or edge trees. In this investigation, CF measurements in both leaves and bark were compared with wood density and the percentage of decay over three seasons (spring, summer and autumn).

### Chlorophyll fluorescence measurements

Chlorophyll fluorescence data were collected and analysed according to the method described in Johnstone *et al.* (2012). Branches ∼10 mm in diameter were harvested from the upper canopy with a 12-gauge shotgun in the morning, between 0600 and 0800 h depending on the season. Leaf fluorescence measurements were taken between 13 September and 21 September 2007 (spring), 28 January and 1 February 2008 (summer) and 5 April and 13 April 2008 (autumn). Most eucalypts can have two or three different leaf ages present in the crown at any one season, with leaves lasting up to 18 months. Eucalypts have opportunistic crown phenology dependent on their environmental conditions (Jacobs 1955).

Leaf CF measurements were taken on mature sun leaves from upper canopy branches using a Hansatech-handy plant efficiency analyser (Hansatech Instruments, King's Lynn, Norfolk, UK). Ten leaves from each tree were dark adapted for 30 min with leaf clips. A saturating flash of red light onto the leaf after the period of darkness induced a time-dependent fluorescence kinetic known as the Kautsky effect (Govindjee 2004; Percival 2005). All trees were tested within 2–3 h of being harvested as recommended by Epron and Dreyer (1992).

Bark CF testing was performed in a 350-mm strip in a cross-section of the trunk on the north half of the trees, 35 mm apart. The test area on the bark was circular and 4.5 mm in diameter. Eight to 10 tests were performed on each tree after material had been dark adapted for 30 min. The bark was not damaged or removed in any way. Test results were excluded if the bark was damaged, decorticating or had only recently been exposed to sunlight. The height at which trees were measured was variable as it was necessary to measure above the sock of rough bark at the base. Bark fluorescence measurements were taken between 24 September and 28 September 2007 (spring), 22 January and 26 January 2008 (summer) and 31 March and 4 April 2008 (autumn).

The CF data were averaged from 8–10 measurements from each tree in each tissue (bark and leaf) and in each season. The ratio *F*_v_/*F*_m_ was calculated from the raw CF data. *F*_v_/*F*_m_ is a derived measure *F*_v_ = *F*_m_ − *F*_o_, where *F*_v_ is the difference between maximum (*F*_m_) and minimum (*F*_o_) CF (Maxwell and Johnson 2000). In addition to calculating the *F*_v_/*F*_m_ ratio, time data taken over a 1-s period were logarithmically transformed and the O–J–I–P CF phases were allocated following the method devised by Strasser and Stirbet (2001).

Each polyphasic increase in fluorescence was characterized by examining logarithmic graphs for each season and in both leaf and bark tissues. After an exponential rise in graphed data, each phase was deemed complete, with the next phase being deemed to start at the critical point (O, J, I or P). Every step is followed by a characteristic temporary decrease or dip (Strasser *et al.* 2004). There was no ‘K’ step observed on the graphs. ‘O’ was at the origin, taken at 0.05 ms, as in many other studies (Krause and Weis 1984; Susplugas *et al.* 2000; Strasser and Stirbet 2001; Govindjee 2004; Strasser *et al.* 2004; Percival 2005). The O–J phase was characterized as ending at 4 ms (J step). The ‘I’ step in leaf fluorescence data was observed at 60 ms and in bark at 90 ms. The ‘P’ step was observed at ∼700 ms on leaf fluorescence graphs, previously observed at 200–300 ms in other studies. The ‘P’ step was not observed in bark fluorescence as the last recording point taken by the instrument was at 1000 ms, and fluorescence was still increasing at this time. The JIP test was not applied to the data; comparisons were made using the raw fluorescence values for O (0.05 ms all data), J (4 ms all data), I (60 ms leaf data, 90 ms bark data), P (700 ms leaf data) and the 1000 ms data point on bark.

### Wood density measurement and wood decay estimation

The 36 *E. saligna* were tested for basic wood density from a small sample collected from the trunk at 1.5 m in height from the trees when they were felled in 2008. Basic wood density was estimated as oven dry mass of wood/volume of wood when ‘green’ (Walker *et al.* 1993). Wood decay in the trees was quantified using the Resi system utilizing the IML-Resi constant feed drill described in Johnstone *et al.* (2007, 2010*b*). The method begins with cross-sectional drilling measurements of the trunk at 0.3 m. The method combines the IML-Resi raw data and Shigo's (1979) compartmentalization of decay in trees (CODIT) model to predict the quantity of wood decay beyond the linear drill locations of the IML-Resi. The method relied on the experienced use of the IML-Resi, knowledge of models of decay in trees and image analysis software (Johnstone *et al.* 2007, 2010*b*).

### Statistical analysis of data

A comparison was made between spring, summer and autumn CF data and wood density and wood decay data using simple linear regression analysis. Simple linear regression analyses were performed using the software package SAS (Statistical Analysis System) version 9.2 (SAS Institute Inc., Cary, NC, USA). Although multiple comparisons were made, Bonferroni corrections were not applied in order to maximize statistical power and minimize Type II errors in the analysis (Moran 2003).

One tree had no leaves and could not be included in leaf CF analysis, and the bark of this tree had died by the autumn sampling date. Data more than two standard deviations away from the next nearest result were considered outliers and eliminated from analysis, resulting in 34–35 individual replicate trees for regression analysis.

## Results

### Comparing leaf and bark fluorescence and basic wood density

**Figure 1.**
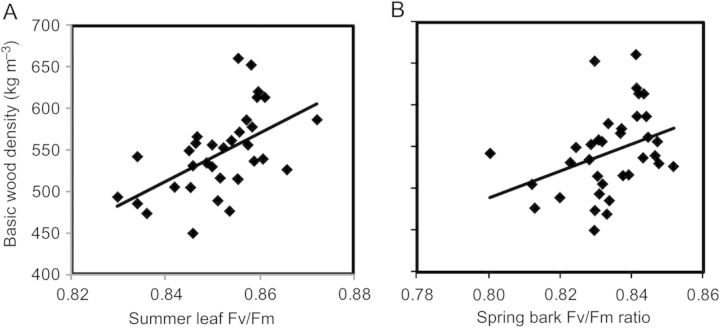
(A) Basic wood density in kg m^−3^ versus summer leaf *F*_v_/*F*_m_. Trend line = linear regression, *P*=0.001, *r*^2^=0.291. *F*_v_/*F*_m_ ratio data begin at 0.820, and basic density data begin at 400 kg m^−3^. (B) Basic wood density in kg m^−3^ versus spring bark *F*_v_/*F*_m_. *F*_v_/*F*_m_ ratio data begin at 0.7900, and basic density data begin at 400 kg m^−3^. Trend line = linear regression, *P* = 0.035, *r*^2^ = 0.128.

There was a statistically significant and positive relationship between summer leaf *F*_v_/*F*_m_ and basic wood density (Table 1 and Fig. 1A). There was a statistically significant and positive relationship between spring bark *F*_v_/*F*_m_ and basic wood density (Table 1 and Fig. 1B). There was no statistical relationship between spring and autumn leaf *F*_v_/*F*_m_ or summer and autumn bark *F*_v_/*F*_m_ and basic wood density (Table 1). There was no statistical relationship between spring, summer and autumn leaf CF at the O, J, I or P step and basic wood density (Table 2). There was also no statistically significant relationship between spring, summer and autumn bark CF at the O, J, I or 1000 ms step and basic wood density (Table 2).

**Table 1.** Summarized results from simple linear regression analyses comparing spring, summer and autumn leaf or bark *F*_v_/*F*_m_ with basic wood density data. *n*, the number of samples; *P*, the probability for the *t*-test that the coefficient of the independent variable is equal to zero; *r*^2^, the variation in the dependent variable that can be explained by the fluorescence data. ^a^The dependent variable is the spring basic wood density data in all cases. ^b^The statistical relationship is significant and positive. Bold values indicate statistical significance.

Independent variable^a^	*n*	*P*	*r*^2^
Spring leaf fluorescence—*F*_v_/*F*_m_	34	0.531	0.012
Summer leaf fluorescence—*F*_v_/*F*_m_	34	**0.001^b^**	**0.291**
Autumn leaf fluorescence—*F*_v_/*F*_m_	35	0.387	0.023
Spring bark fluorescence—*F*_v_/*F*_m_	35	**0.035^b^**	**0.128**
Summer bark fluorescence—*F*_v_/*F*_m_	35	0.512	0.013
Autumn bark fluorescence—*F*_v_/*F*_m_	35	0.249	0.040

**Table 2.** Summarized results from simple linear regression analyses comparing spring, summer and autumn leaf or bark net chlorophyll fluorescence with basic wood density data. Results from these analyses were not significant. *n*, the number of samples; *P*, the probability for the *t*-test that the coefficient of the independent variable is equal to zero; *r*^2^, the variation in the dependent variable that can be explained by the fluorescence data. ^a^The dependent variable is the spring basic wood density data in all cases.

Independent variable^a^	*n*	*P*	*r*^2^
Spring leaf fluorescence—‘O’ step	34	0.741	0.004
Spring leaf fluorescence—‘J’ step	34	0.620	0.008
Spring leaf fluorescence—‘I’ step	34	0.462	0.017
Spring leaf fluorescence—‘P’ step	34	0.891	0.001
Spring bark fluorescence—‘O’ step	35	0.702	0.005
Spring bark fluorescence—‘J’ step	35	0.691	0.005
Spring bark fluorescence—‘I’ step	35	0.298	0.033
Spring bark fluorescence—1000 ms	35	0.173	0.056
Summer leaf fluorescence—‘O’ step	34	0.072	0.097
Summer leaf fluorescence—‘J’ step	34	0.085	0.090
Summer leaf fluorescence—‘I’ step	34	0.134	0.069
Summer leaf fluorescence—‘P’ step	34	0.913	0.000
Summer bark fluorescence—‘O’ step	35	0.309	0.031
Summer bark fluorescence—‘J’ step	35	0.832	0.001
Summer bark fluorescence—‘I’ step	35	0.256	0.039
Summer bark fluorescence—1000 ms	35	0.191	0.051
Autumn leaf fluorescence—‘O’ step	34	0.810	0.002
Autumn leaf fluorescence—‘J’ step	34	0.558	0.011
Autumn leaf fluorescence—‘I’ step	34	0.905	0.001
Autumn leaf fluorescence—‘P’ step	34	0.747	0.003
Autumn bark fluorescence—‘O’ step	35	0.427	0.020
Autumn bark fluorescence—‘J’ step	35	0.461	0.017
Autumn bark fluorescence—‘I’ step	35	0.734	0.004
Autumn bark fluorescence—1000 ms	35	0.828	0.002

### Comparing leaf and bark fluorescence and wood decay

**Figure 2.**
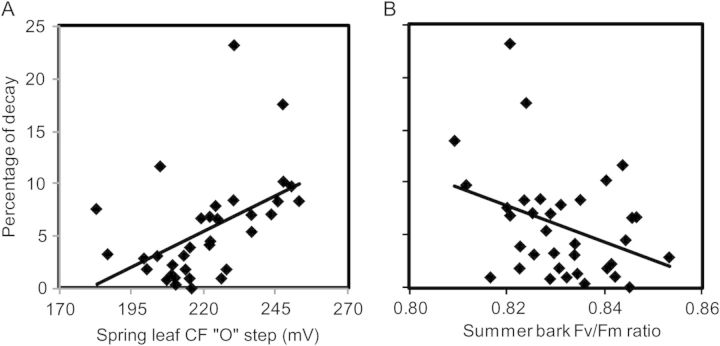
(A) Percentage of decay using the Resi system versus spring leaf chlorophyll fluorescence at the ‘O’ step in millivolts. Chlorophyll fluorescence data begin at 100 mV. Trend line = linear regression, *P* = 0.004, *r*^2^ = 0.230. (B) Percentage of decay using the Resi system versus summer bark *F*_v_/*F*_m_. *F*_v_/*F*_m_ ratio data begin at 0.800. Trend line = linear regression, *P* = 0.021, *r*^2^ = 0.148.

There was a statistically significant and positive relationship between spring leaf CF at the O step and wood decay (Table 3 and Fig. 2A). There was a statistically significant and negative relationship between the summer leaf *F*_v_/*F*_m_ ratio and wood decay (Table 3). There was a statistically significant and negative relationship between spring, summer and autumn bark *F*_v_/*F*_m_ and wood decay (Table 3 and Fig. 2B).

**Table 3.** Summarized results from simple linear regression analyses comparing spring, summer and autumn leaf or bark *F*_v_/*F*_m_ and ‘O’ step fluorescence values with wood decay data. *n*, the number of samples; *P*, the probability for the *t*-test that the coefficient of the independent variable is equal to zero; *r*^2^, the variation in the dependent variable that can be explained by the fluorescence data. ^a^The dependent variable is wood decay in all cases. ^b^The statistical relationship is significant and positive. ^c^The statistical relationship is significant and negative. Bold values indicate statistical significance.

Independent variable^a^	*n*	*P*	*r*^2^
Spring leaf fluorescence—*F*_v_/*F*_m_	34	0.505	0.014
Spring leaf fluorescence—‘O’ step	34	**0.004^b^**	**0.230**
Spring bark fluorescence—*F*_v_/*F*_m_	35	**0.036^b^**	**0.127**
Spring bark fluorescence—‘O’ step	35	0.363	0.025
Summer leaf fluorescence—*F*_v_/*F*_m_	34	**0.025^c^**	**0.148**
Summer leaf fluorescence—‘O’ step	34	0.080	0.093
Summer bark fluorescence—*F*_v_/*F*_m_	35	**0.037^b^**	**0.125**
Summer bark fluorescence—‘O’ step	35	0.101	0.079
Autumn leaf fluorescence—*F*_v_/*F*_m_	35	0.853	0.001
Autumn leaf fluorescence—‘O’ step	34	0.870	0.001
Autumn bark fluorescence—*F*_v_/*F*_m_	35	**0.034^b^**	**0.129**
Autumn bark fluorescence—‘O’ step	35	0.363	0.025

There was no statistically significant relationship between the spring leaf *F*_v_/*F*_m_ ratio or CF at the J, I and P step and wood decay (Table 4). There was no statistically significant relationship between summer leaf CF at the O, J, I, and P step and wood decay (Table 4). There was no statistically significant relationship between autumn leaf CF and wood decay (Table 4). There was no statistically significant relationship between bark CF at the O, J, I or 1000 ms step and wood decay, in spring, summer or autumn (Table 4).

**Table 4.** Summarized results from simple linear regression analyses comparing spring, summer and autumn leaf or bark ‘J’, ‘I’, ‘P’ or 1000 ms fluorescence values with wood decay data. Results from these analyses were not significant. *n*, the number of samples; *P*, the probability for the *t*-test that the coefficient of the independent variable is equal to zero; *r*^2^, the variation in the dependent variable that can be explained by the fluorescence data. ^a^The dependent variable is the wood decay data in all cases.

Independent variable^a^	*n*	*P*	*r*^2^
Spring leaf fluorescence—‘J’ step	34	0.076	0.095
Spring leaf fluorescence—‘I’ step	34	0.456	0.018
Spring leaf fluorescence—‘P’ step	34	0.158	0.062
Spring bark fluorescence—‘J’ step	35	0.207	0.048
Spring bark fluorescence—‘I’ step	35	0.617	0.008
Spring bark fluorescence—1000 ms	35	0.901	0.001
Summer leaf fluorescence—‘J’ step	34	0.104	0.081
Summer leaf fluorescence—‘I’ step	34	0.452	0.018
Summer leaf fluorescence—‘P’ step	34	0.660	0.006
Summer bark fluorescence—‘J’ step	35	0.095	0.082
Summer bark fluorescence—‘I’ step	35	0.295	0.033
Summer bark fluorescence—1000 ms	35	0.430	0.019
Autumn leaf fluorescence—‘J’ step	34	0.969	0.000
Autumn leaf fluorescence—‘I’ step	34	0.350	0.027
Autumn leaf fluorescence—‘P’ step	34	0.319	0.031
Autumn bark fluorescence—‘J’ step	35	0.478	0.015
Autumn bark fluorescence—‘I’ step	35	0.691	0.005
Autumn bark fluorescence—1000 ms	35	0.987	0.000

## Discussion

Weight loss or its corollary wood density has been used to assess wood decay for many years (Kennedy 1958; Wilcox 1978; Wei *et al.* 2010). Wood decay organisms can be responsible for weight losses as small as 5 % or less (Noguchi *et al.* 1986). In instances of very early decay, even a light microscope may not be able to detect wood decay visually (Wilcox 1978). Hence there is clearly a strong relationship between measured wood density and wood decay, even in assumed sound or intact wood.

There was a statistically significant and positive relationship between summer leaf *F*_v_/*F*_m_ and basic wood density, but not in spring or autumn. In this study, the summer period of investigation coincided with maximum seasonal tree stress in southern Australia, when the mean average maximum temperature at the test site in January 2008 was 27 °C (minimum average 16 °C, Bureau of Metrology Australia 2008). However, summer predawn leaf and stem water potentials were not significantly different from spring values, although values of around 1.2 MPa for all trees in summer indicated mild to moderate drought stress at this site (White *et al.* 2000; Johnstone *et al.* 2012). Predawn water potentials did not show a relationship with wood density or decay, but water potentials were not measured during the day when water deficit becomes more noticeable. Summer leaf CF at the O step also correlated with a visual vitality measurement in summer (Johnstone *et al.* 2012), which suggests that the trees were suffering some type of stress during the seasonal summer drought and that the leaf O step was sensitive to the stress. Wood density is sometimes measured in conjunction with other parameters for assessing the water status of trees (O'Grady *et al.* 2009; Gotsch *et al.* 2010). Low stem wood density in angiosperms is sometimes thought to be indicative of increased vulnerability to xylem cavitation during drought stress (Holste *et al.* 2006; Bobich *et al.* 2010). If cavitation is occurring in the xylem of the *E. saligna* in the current study, it could establish a favourable environment for fungal pathogens (Rayner and Boddy 1988), or the pathogens may assist with the cavitation process (Tyree and Sperry 1989; Tyree and Zimmermann 2002). This may explain why there is a relationship between leaf CF and wood decay in two seasons (spring and summer) rather than just one as was the case with wood density.

Changes in the availability of water for the *E. saligna* may have contributed directly to the relationship between photosynthetic efficiency and wood decay discovered in this study. The water saturation of wood has long been known to prevent the development of wood decay, and air is necessary for the development of decay in wood (Rayner and Boddy 1988). The barrier zones in Shigo's CODIT model (Shigo 1979) are said to be a response to xylem embolism by Rayner and Boddy (1988), rather than the incursion of decay organisms *per se*. Cavitation during moisture stress is one way a xylem vessel can develop an embolism (Tyree and Sperry 1989). The direct relationship between xylem cavitation and wood decay has not been evaluated, but the introduction of a gaseous phase during the compartmentalization process, according to Rayner and Boddy (1988), is a primary component in the development of wood decay in trees. It is when tree wood dries out that compartmentalization barriers are breached (Rayner and Boddy 1988).

Trees with lower wood density have also been associated with an increased risk of cavitation (Holste *et al.* 2006; Bobich *et al.* 2010). Therefore, it is not surprising that in this study, in the hot Australian summer, *E. saligna* showed an inverse relationship between leaf CF and wood density, and an even stronger relationship between leaf CF and wood decay. Unlike many other studies, the link between moisture stress, cavitation, embolism and wood density/decay described here is a within-species effect, rather than the ecological inter-species effect of low wood density and water relations/growth discussed in other studies (Bucci *et al.* 2004; O'Grady *et al.* 2009). The lower density wood is produced due to stressful environmental conditions or is a result of very early wood decay in the *E. saligna*; there are no genetic differences or predispositions at play. The link between wood density, leaf CF and wood decay within species established in this study has not been previously reported.

There was a statistically significant and positive relationship between leaf CF values at the ‘O’ step and wood decay in spring. The O–J step is believed to represent the reduction of the plastoquinone Q_A_ molecule from Q_A_ to Q_A_^−^ between PSII and photosystem I (PSI) (Hsu and Leu 2003; Strasser *et al.* 2004; Percival 2005); therefore, it appears that the reduction of Q_A_ between PSII and PSI during leaf photosynthesis is associated with wood decay in *E. saligna. F*_v_/*F*_m_ is the theoretical measure of the quantum efficiency of PSII if all the PSII reaction centres are open (Maxwell and Johnson 2000). There was a significant and negative relationship between the leaf CF *F*_v_/*F*_m_ ratio and wood decay in summer, suggesting that wood decay may also be associated with the quantum efficiency of PSII in leaves.

This study further emphasizes the link between the operation of photosynthesis in leaves and environmental stress. The O step in the OJIP fluorescence transient in leaves, which relates to the part of the photosynthetic light reaction where plastoquinone Q_A_ electron carrier molecules are in their oxidized state between PSII and PSI, is particularly affected by moisture stress in other studies of trees (Epron *et al.* 1992; Percival and Sheriffs 2002). This study establishes a new link between the quantum efficiency of PSII (*F*_v_/*F*_m_) in leaves, wood density and wood decay. The study also establishes a new and consistent pattern of correlation between the quantum efficiency of PSII (*F*_v_/*F*_m_) in bark and environmental stress, wood decay and to a lesser extent wood density. Further research could examine the link between the quantum efficiency of PSII in bark in relation to other tree species, and other environmental stressors.

Christen *et al.* (2007) investigated the esca disease in *V. vinifera* (grapevines) and the relationship between the white rot decay and/or necrosis of woody tissues, the wilting of leaves and CF parameters. They used four categories of white rot decay and eight categories of necrosis, rather than percentages of decay. Necrosis and white rot were more widespread in Cabernet Sauvignon than in Merlot plants. The more decayed Cabernet Sauvignon plants showed decreased efficiency in PSII and the PI_ABS_ (performance index) value according to the CF results, compared with the Merlot population. However, the statistical relationship between CF and wood decay was only significant at a cultivar level in *V. vinifera*, rather than at an individual plant level, as was the case in the *E. saligna* from the current study.

The question as to why trees with decreased photosynthetic efficiency are more decayed is not easy to answer. Lorio (1986) suggested that the production of oleoresin, a protective agent against *Dendroctonus frontalis* (southern pine beetle) in *Pinus taeda* (loblolly pine), is lower in suppressed trees when the production of wood is depressed. Hence, one reason why *E. saligna* trees with decreased photosynthetic efficiency may be more decayed may be because when the growth of wood is depressed, the synthesis of protective chemical compounds produced in the wood is also decreased. In addition, research on the progression of wood decay in trees suggests that the origin of wood decay can be in the sapwood, rather than from saprotrophic growth in non-functioning heartwood (Boddy and Rayner 1983; Parfitt *et al.* 2010). It is possible that during the seasonal challenge inherent in hot summers, there is pressure on photosynthesis as a result of stomatal closure and the resultant high light stress on photosystems (Faria *et al.* 1998). Therefore, under such conditions trees with partly decayed primary xylem suffer more because they may have to close their stomata more or more often because their water transport system is less efficient.

Induction curves did not reach their maximum in the bark fluorescence measurements; however, high *F*_v_/*F*_m_ ratios indicate that values were close to maximum and were not significantly biased by low light intensities (Johnstone *et al.* 2012). Bark *F*_v_/*F*_m_ ratios were negatively correlated with wood density in *E. saligna* in spring only, and in this instance the bark CF statistical relationships were weaker than those for leaf CF (Tables 1 and 2). The statistical relationships between bark *F*_v_/*F*_m_ ratios and wood decay were weak but more consistent over three seasons than correlations with leaf parameters (Tables 3 and 4). Therefore, PSII in leaves may be more sensitive to the immediate effects of water flow disruption than bark photosynthesis, but the longer-term sustained effects of moisture stress, such as cavitation and the subsequent entry of wood decay pathogens, affect PSII in bark in a more consistent pattern than in the leaves. Stem photosynthesis is believed to use gaseous xylem efflux as a source of CO_2_ (Pfanz 2008); therefore, if the xylem is not fully functioning it may affect the health of bark chlorenchyma, and thus PSII. Some tree species have been found to have elevated CO_2_ in decayed wood tissues, while CO_2_ was depressed in winter in other species (Rayner and Boddy 1988); thus the complex interactions of the metabolism of xylem, bark and wood decay organisms warrant further investigation.

The transpirational xylem stream supplies inorganic nutrients (and water) to bark chlorenchyma (Pfanz 2008), so if the xylem stream is disrupted it may affect stem photosynthesis. *Eucalyptus* sp. may be sensitive to factors that affect stem photosynthesis as stem photosynthesis may be a more important source of photosynthates for them than for other broadleaved trees, because they have a low leaf area index and are prone to defoliation by insects, diseases or drought (Tausz *et al.* 2005; Eyles *et al.* 2009). Interestingly, unlike the leaf CF measurements, only the quantum efficiency (*F*_v_/*F*_m_) of PSII within bark chlorenchyma was associated with wood decay; the reduction of the plastoquinone Q_A_ molecule between PSII and PSI (O–J step) was not affected.

The PI_ABS_ CF value has been used to successfully quantify drought stress in trees (Percival and AlBalushi 2007; Swoczyna *et al.* 2010). The PI_ABS_ value was not calculated in the current study, as it is not as widely used as the *F*_v_/*F*_m_ value. Future studies could examine the effect of wood decay and wood density in trees on the PI_ABS_ value in relation to bark photosynthesis and other derived measures that form part of the ‘JIP test’, such as the apparent rates of photosynthetic electron transport and non-photochemical quenching (Lüttge *et al.* 2003).

## Conclusions

The CF measurements in this study clearly support the hypothesis that there is a relationship between CF and wood structural changes. The results suggest that when photosynthesis is impaired, trees are more prone to wood decay and low wood density. Although chlorenchymes are present in bark and indeed in many woody tree tissues (Pfanz *et al.* 2002), it may not be possible to test trunk tissue in many tree species due to peridermal thickening (Aschan *et al.* 2001). However in some studies photosynthesis has been measured successfully in stems (Damesin 2003; Wittmann and Pfanz 2008*a*). This raises the possibility of testing larger branches on many temperate species where the bark is not as thick as on the trunk of the tree. This study showed that the reduced functioning of PSII in bark chlorenchyma in particular is an indication that a tree may have a larger quantity of decay in the xylem tissues.

## Sources of Funding

Our work was funded by the University of Melbourne, Australia.

## Contributions by the Authors

D.J. performed all of the collection and analysis of data, was responsible for the experimental design and wrote most of the introduction, methods, results, discussion and conclusion. M.T. assisted in the design of the experiments and wrote sections of the introduction and discussion. G.M. offered advice on experimental design and assistance with the introduction and discussion sections. M.N. contributed to the experimental design.

## Conflicts of Interest Statement

None declared.
